# Advances in Cognitive Remediation Training in Schizophrenia: A Review

**DOI:** 10.3390/brainsci12020129

**Published:** 2022-01-18

**Authors:** Brianna Fitapelli, Jean-Pierre Lindenmayer

**Affiliations:** 1 Nathan S. Kline Institute for Psychiatric Research, Orangeburg, NY 10962, USA; Jean-Pierre.Lindenmayer@omh.ny.gov; 2 Manhattan Psychiatric Center, New York, NY 10035, USA; 3 Department of Psychology, Teachers College, Columbia University, New York, NY 10027, USA; 4 School of Medicine, New York University, New York, NY 10012, USA

**Keywords:** cognitive remediation, schizophrenia, cognitive functioning, cognitive training

## Abstract

Cognitive Remediation Training (CRT) in schizophrenia has made great strides since its introduction in the 1990s. CRT was developed with the aim of improving the everyday functioning of individuals living with cognitive impairment. MEDLINE, PsychINFO, and Google Scholar were searched to extract peer-reviewed randomized controlled trials to produce the current review article. The aim of the present review is to summarize CRT effects on addressing cognitive changes in patients undergoing CRT as defined by the Cognitive Remediation Experts Workshop and to describe the areas of greatest impact in specific cognitive domains. Another area of this review aims to summarize the modalities of intervention (paper and pencil; computerized; home bound), the persistence of improvements, and their generalization to other domains of functioning. Finally, this review delineates barriers for wider dissemination of CRT, such as the transfer of research findings into clinical everyday practice and future developments of CRT.

## 1. Introduction

Cognitive impairment is a core feature of schizophrenia. Evidence has shown a strong association between cognitive impairment and functioning in daily life, such as employment, interpersonal relationships, and independent living [1]. Further, individuals with impaired cognitive functioning may also struggle to adequately respond to various forms of rehabilitation that aim to improve self-care, social skills, and job skills [1]. Cognitive deficits can impede nearly all aspects of life and are therefore an important target for further study and treatment [1]. Psychopharmacological interventions generally do not address cognitive dysfunctions, hence the need for alternative interventions [2,3].

Cognitive remediation training (CRT) is an evidence-based treatment that has been shown to produce improvements in cognition in individuals with schizophrenia [1,4]. When CRT is combined with other forms of psychiatric rehabilitation, improvements in functional outcomes have also been shown [4]. According to the Cognitive Remediation Experts Workshop, cognitive remediation therapy for schizophrenia is “a behavioral training-based intervention that aims to improve cognitive processes (attention, memory, executive function, social cognition, or metacognition) with the goal of durability and generalization [5].” However, improvements seen with CRT have varied, due to a number of implementation and measurement factors [1], the targeted patient population [6] and intrinsic limitations in generalizability [5]. There has been a large variability in how CRT is implemented (paper/pencil, computerized, error-free learning), in the different cognitive outcomes targeted, and in different functional and long-term outcomes measured [1,4,5,6]. Another factor contributing to the variability in reported results may be due to the degree that targeted interventions transfer what was learned in CRT to real world cognitive tasks and to the inclusion of meta-cognitive goals in CRT [3,4].

Although there have been a number of methodologically rigorous meta-analyses completed over the course of the past decade regarding CRT in schizophrenia, new data has since emerged that has not been previously reviewed. Moreover, the scope of our review covers numerous aspects of CRT, some of which were not examined in previous meta-analyses that utilized a narrower approach. The aims of the present review are to (1) review the modalities of intervention (paper and pencil; computerized; group format; home bound); (2) to examine CRT results in terms of cognitive changes in patients undergoing CRT, to describe the areas of greatest cognitive impact together with predictors of response, and to examine the persistence of improvements and their generalization to other domains of functioning; and (3) to delineate barriers and promises in further development of CRT.

## 2. Materials and Methods

**Figure 1:**
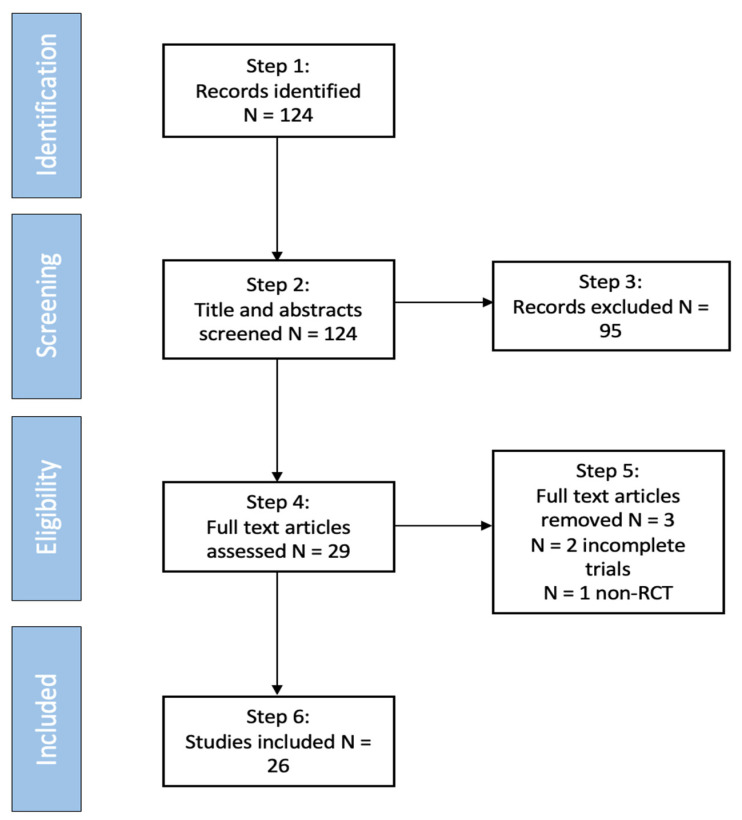
Consort flow diagram representing articles included and excluded in the review process.

Studies for this review were identified by conducting MEDLINE, PsychINFO, and Google Scholar searches for articles published in peer-reviewed journals. Articles from January 2011 to October 2021 were considered for this review. The following search terms and keywords were used: cognitive remediation, cognitive training, and schizophrenia. The references of the studies found were hand-searched for other relevant studies that fulfilled our inclusion criteria as follows: (1) randomized-controlled trials (RCTs) fulfilling the Cognitive Remediation Experts Working Group definition for cognitive remediation [5], which included individuals with schizophrenia and/or schizoaffective disorder, (2) studies that were written in English or had a published English translation, (3) studies that included participants that were adults over 19 years of age, (4) studies that were published in peer-reviewed journals, and (5) studies that included a full battery of baseline and endpoint-administered cognitive tests. Our search resulted in 124 studies (see Figure 1) covering both in- and outpatients and were checked for relevance with the above criteria. A total of 95 publications were removed after a review of the titles and abstracts, because they did not fulfill our inclusion criteria due to their non-RCT design, inclusion of individuals under 19 years of age, lack of systematic cognitive assessments administered, or the study had been published before 2011. The remaining 29 studies were examined in full-length based on our three stated aims of reviewing the methodology, efficacy, and long-term effects of CRT. Three studies were further removed after a full examination of the text: two trials were not completed and the other was not an RCT. The remaining 26 studies included in the review are presented in detail in Table 1.

## 3. Results

A total of 26 studies, with 1646 participants, fulfilled all inclusion criteria (see Table 1). Study samples consisted of individuals with a mean age of 37.29 (*SD* = 6.69). The mean percentage of males was 68.30 (*SD* = 11.41) and that of females was 31.70 (*SD* = 11.41). Of the 26 studies, 10 were outpatient studies, 11 included inpatients, and 5 included both inpatients and outpatients. The mean duration of cognitive remediation interventions was 11.23 weeks (*SD* = 5.35) with a total mean number of sessions of 31.97 (*SD =* 17.41). The mean duration of individual sessions was 76.77 min (*SD =* 35.98).

### 3.1. CRT Methodologies

CRT programs of late have shifted from the traditional paper-and-pencil format to a computerized format. Out of the 26 studies included, two included a paper-and-pencil format that had dropout rates of 0 [9] and 8 [15]. Twenty-three studies included a computerized CRT program with dropout rates ranging from 0 to 53 [7,8,10,11,12,13,14,16,17,18,19,20,21,22,23,24,26,27,28,29,30,31,32]. One study compared paper-and-pencil and computerized CRT to one another and had 9 total dropouts [25]. It is unclear from these results if computerized CRT programs are superior to paper-and-pencil. In two meta-analyses, Wykes et al. (2011) and Vita et al. (2021) found no effect on cognitive outcomes with computer-assisted CRT programs compared with traditional approaches [6,33].

Both group and individual formats implementing CRT were found from our search. Of the 26 articles, nine studies used an individual format [7,11,12,13,14,23,24,29,31] and 17 used a group format [8,9,10,15,16,17,18,19,20,21,22,25,26,27,28,30,32]. One study [22] had participants complete homework exercises in their own homes, but the CRT program itself was implemented in a group outpatient setting. Group formats may be advantageous considering that fewer staff are needed to facilitate a group of patients compared with an individual format, thereby reducing the staffing cost of CRT administration. Group facilitation, given the proper training and supervision of group leaders, may also benefit the participant’s awareness of others and motivation to complete the task at hand.

#### Bottom-Up versus Top-Down Approaches

As most CRT programs are considered to use “top-down” approaches, it is not surprising that approximately 75% of the studies in our review followed this format. Top-down approaches to CRT target higher-order neurocognitive operations, such as working memory, strategy learning, and problem-solving [34]. It is proposed that focusing on these higher order executive functions can induce functional and structural brain changes (i.e., increased activation in the prefrontal cortex) by training more complex abilities such as attention and speed of processing [34]. Bottom-up approaches are less common but are gaining much more attention of late. These neuroplasticity-based interventions help to train perceptual processes while concurrently engaging working memory and attentional operations [35]. Throughout prefrontal-temporo-parietal systems, bottom-up approaches are designed to drive adaptive plastic change. For example, intensive auditory or visual training within CRT can improve perceptual abilities and generate restoration of prefrontal functions and higher-order cognition [34,36]. Examples of studies that have implemented both approaches are further described below.

### 3.2. Moderating Variables

#### 3.2.1. Bridging Groups

Bridging groups are groups focusing on the transfer and application of cognitive skills learned in CRT to everyday living situations and were implemented in four of the 22 studies. Typically, bridging groups are coordinated by a trained therapist who facilitates the process of the group. In addition to the CRT intervention, two studies [18,21] implemented a separate group session that met once per week for 12 weeks. The groups were designed to facilitate connections between a computerized CRT program and work performance or daily life, and to assist in tracking and setting individualized goals for life and work within the community. These groups included a discussion on what tasks participants found easy or difficult within the CRT program and the strategies that could be used to complete the tasks. Preparatory conversations regarding community living were discussed, as well as how the role of cognition is incorporated into job performance, and compensatory strategies for challenges that often arise within the workplace. Both studies used strategies from the “Thinking Skills for Living Group” method [37,38,39].

The remaining three studies [26,28] that implemented bridging groups utilized the “Neuropsychological Educational Approach to Remediation” (NEAR) to teach participants how to apply the cognitive skills acquired from the CRT program to daily tasks, to promote group identity and to promote socialization. In all the above studies, bridging groups were highly structured with detailed manuals, and were facilitated by trained and qualified staff. Bridging groups have been shown to ameliorate the transfer of cognitive gains from CRT programs into real-world settings, more so than purely drill-and-practice strategies alone This last point is further confirmed by a recent meta-analysis by Vita et al. (2021), which found that CRT conducted with trained therapists is clearly more efficacious than CRT conducted without a trained therapist [33].

#### 3.2.2. The Role of Metacognition in CRT

Rather than a drill-and-practice approach alone, several studies used a drill-and-strategy-based approach to enhance metacognition. Metacognition is cognition of one’s own awareness and understanding of one’s own thought process [4]. CRT that incorporates strategies that help participants understand the abstract principles underlying specific tasks has been shown to be superior in comparison to drill-and-practice strategies alone [4]. For example, one study [18] developed an original CRT program by adapting COGPACK to meet the needs of a Japanese population called JCORES, or the Japanese Cognitive Rehabilitation Program. While participants were partaking in the cognitive exercises, therapists guided the process by asking questions aimed at enhancing metacognition and information processing. Such a strategy-based approach was implemented in several other studies that included performance feedback, encouragement to practice new strategies in future sessions, and compensatory strategies relevant to the individual’s needs [9,15,16,22,26,28,30,31,32]. One study allotted 10 min before and after each CRT session for participants to socially interact and share learning strategies [25]. Although the authors did not follow the stringent procedures of a bridging group, a significant improvement in overall cognition and psychosocial functioning was found.

#### 3.2.3. The Role of Adding Other Rehabilitative Interventions

Many CRT studies were conducted in settings where CRT was embedded in a more comprehensive rehabilitative setting. This embeddedness provides opportunities for participants to practice new cognitive skills and to reinforce their acquisition through practical tasks of daily living [1]. The beneficial moderating effect of embedding CRT into a rehabilitation program has been further confirmed by the recent meta-analysis from Vita et al. (2021) where a significant effect on functioning was found [33]. Further, Bowie et al. (2012) compared functional skills training alone to cognitive remediation alone and to a combination of the two interventions. Only when CRT was provided was there a benefit noted. However, the strongest effect was found when the two therapies were provided together [40]. Similarly, McGurk et al. (2009) examined the effects of adding CRT to a vocational services program and found significantly greater improvements in cognition over 3 months and better work outcomes at a 2-year follow-up in the CRT plus vocational services program compared with vocational services alone [38].

CRT programs have also been augmented by adding other targeted interventions to CRT, such as social skills and social cognition training. Two studies combined CRT with a social cognition training program called MRIGE and compared it to CRT alone, showing significant improvements in emotion perception, emotion recognition, and greater gains in neurocognition as compared to CRT alone [10,21]. Three studies [15,19,30] utilized a cognitive CRT program, REHACOP, which combined cognitive remediation, social cognitive intervention, and functional skills training all embedded within the computerized software. The authors found significant improvements in social cognition, neurocognition, and functioning in the REHACOP group as compared to the control [15,19,30]. Kurtz et al. (2015) augmented a social skills training program with CRT and found significant improvements in attention, working memory, and social functioning, specifically in empathy [17]. These results suggest that the addition of social skills or social-cognitive training with CRT will augment the cognitive domains that contribute to improved social functioning related to the understanding of another person’s emotions, feelings, and perspectives.

Few double-blind studies have examined pharmacological augmentation of CRT, although a case can be made for the synergistic effects of a biological intervention together with a cognitive practice effect [41]. Stimulant medications have the potential to improve attention and processing speed, which in turn may increase participants’ ability to concentrate on cognitive training tasks [41]. Combining cognitive remediation with pharmacological compounds has been termed as Pharmacologically Augmented Cognitive Therapies (PACT) by Swerdlow (2011) [42]. Michalopoulou et al. (2015) reported on a trial using modafinil (200 mg; a wakefulness-promoting medication for narcolepsy) as the pharmacological augmenting agent in 49 participants with chronic schizophrenia in a double-blind, placebo-controlled study [43]. All participants engaged in a concomitant cognitive training program for 10 consecutive days. The primary outcome measure was the performance of the trained tasks, and secondary outcome measures included the MATRICS cognitive battery. There were no differences found between the two groups in terms of cognitive measures [43]. In contrast, Swerdlow et al. (2011) treated patients with schizophrenia with 10 mg of amphetamine or placebo in a double-blind cross-over design before and after 60 min of auditory training [42]. Compared to placebo, amphetamine treatment had a substantial benefit on gains during auditory training, suggesting that session-by-session administration of cognition-enhancing compounds can lead to greater attentional gains with CRT.

Augmentation of CRT with anti-psychotic medications has been tested by Kantrowitz et al. (2016), who reported on a multicenter, rater-blinded, randomized, controlled study of auditory-focused cognitive remediation (BrainFitness) combined with lurasidone (40 to 80 mg daily) of 120 outpatients with schizophrenia [44]. Auditory processing cognitive remediation combined with lurasidone did not lead to differential improvement over lurasidone and nonspecific video games.

More recently, several research compounds with a partial agonistic effect on N-methyl-d-aspartate glutamatergic receptors and alpha-7 nicotinic agonist are being tested as augmentations of CRT, however, results are modest [45].

Finally, CRT has been combined with direct neuro-modulatory interventions, such as concurrent transcranial direct current stimulation (tDCS), to enhance participants’ cognitive gains [46]. Andrews et al. (2011) applied tDCS to the left dorsolateral prefrontal cortex (DLPFC), which has been previously found to improve working memory, in 10 healthy participants. Those who received tDCS plus cognitive training with a working memory task showed a greater improvement in performance compared with sham tDCS and active tDCS alone [46]. These results provide promising data on the benefits of neuro-modulatory interventions with CRT; however, further research is needed for individuals with schizophrenia.

### 3.3. Efficacy of CRT

Three meta-analyses [1,6,33] found moderate improvements in neuropsychological test performance with 12,106 participants in their mid-thirties undergoing CRT for 12.8 to 16.7 weeks. McGurk et al. (2007) found CRT to be associated with significant improvements in cognitive performance with a medium effect size (*d* = 0.41), psychosocial functioning (*d* = 0.36), and symptomatology (*d* = 0.28) [1]. Wykes et al. (2011) found CRT to have durable effects on global cognition and functioning, yet symptom effects were small and not sustained [6]. Vita et al. (2021) found CRT to be effective on global cognition (*d* = 0.29) and functioning (*d* = 0.22) with small effect sizes [33]. When studies provided adjunctive psychiatric rehabilitation and strategic approaches to CRT, psychosocial functioning and cognition showed greater improvements [1,6,33]. However, hours of training, duration, and computer use were not associated with overall cognitive outcomes.

From our review, composite scores of baseline-to-endpoint neurocognitive assessments revealed 11 studies with significant but heterogenous improvements, with effects sizes ranging from *d* = 0.19 to 1.23 [9,10,11,12,16,19,21,22,32]. Two studies did not publish effect sizes, but composite scores were significant when compared to the control group, measured by the Brief Assessment of Cognition in Schizophrenia (BACS; *F* = 4.11, *p* = 0.047) [18] and the MATRICS Consensus Cognitive Battery (MCCB; *F* = 11.50, *p* = 0.002) [23]. Of the 11 studies, seven administered the full MCCB [10,11,12,16,21,22,23], two utilized the BACS [18,32], and one study measured cognition with the Rey Auditory Verbal Learning Test (RAVLT), Wisconsin Card Sorting Test (WCST), the Rey-Osterrieth Complex Figure Test (ROCF), Trial Making Tests Part A and B (TMT-A/B; a measure from the MCCB), and the Auditory Consonant Trigrams (ACT) [9]. The final study [19] administered the Accentuation Reading Test, the Stroop Test, and three measures from the MCCB: the Hopkins Verbal Learning Test (HVLT) and the Digit Span and Digit Symbol subtests from the Wechsler Adult Intelligence Scale-III (WAIS-III). Lu et al. (2012) administered one cognitive battery, the WCST, and found a statistically significant difference (*p* = 0.019) between the control and CRT condition [13].

For improvement in specific cognitive domains, five studies showed improvements in verbal learning, with effect sizes ranging from 0.88 to 1.55 [7,15,32]. Two studies reported significant improvements in verbal learning but did not publish effect sizes: (*p* = 0.020) [9] and (*p* = 0.003) [23]. Working memory improved in nine studies that reported effect sizes [7,10,15,21,22,24,25,29,30] ranging from 0.32 to 0.88. Five studies found improvements in verbal working memory, with effect sizes ranging from *d* = 0.52 to 1.04 [7,14,30] and statistically significant outcomes of (*p* = 0.023) [9] and (*p* = 0.008) [18]. Visual learning was improved in two studies [21,25] with effect sizes ranging from *d* = 0.88–1.51. Gharaeiour et al. (2012) also found a significant improvement in visual learning (*p* = 0.014). Visual memory, measured by the Neuropsychological Test Automated Battery, was improved in one study [31] with effect sizes ranging from 0.14 to 0.71 on various subscales of the measure.

Six studies [10,15,16,21,24,30] reported significant effects on speed of processing ranging from 0.27 to 1.93, and two studies [9,20] reported a statistically significant improvement in speed of processing (*p* = *0*.013 and *p* = *0*.010). Four studies found significant effect sizes on attention ranging from 0.26 to 0.90 [7,10,17,29]. Gharaeipour and Scott (2012) reported a significant improvement in attention assessed by Part A (*p* = 0.044) and Part B (*p* = 0.013) of the Trail Making Test (TMT) but did not report effect sizes [9].

In summary, our results showed moderate improvements in overall global cognition. Of the various cognitive domains examined, working memory benefited most from CRT in 35% of studies reporting significant improvements. Speed of processing improved in 23% of the studies reviewed. Attention, verbal learning, and verbal working memory improved in 19% of studies. Visual learning and memory improved in only 0.03–0.07% of studies. Finally, problem solving and reasoning did not improve in any studies in the present review.

#### 3.3.1. Persistence of Cognitive Improvements

After completion of CRT, five of the 26 studies included longitudinal follow-ups ranging from 3 months [7,11,22,23,26]. All but one study [11] observed sustained improvements in neurocognition. Sustained improvements in functional work outcomes [23] and social cognition [23] and functioning [26] were also shown. Of note, Ventura et al. (2019) implemented CRT for six months and then administered booster sessions for another six months [26]. Booster sessions can be a beneficial strategy to produce larger and more robust improvements in neurocognition and social and functional outcomes over time. There is a great need for future studies to implement longitudinal trials, as the ultimate goal of CRT is to improve cognitive functions in a sustained fashion. In addition, short-term trials may not be adequate to ascertain how cognitive gains will transfer to daily life.

#### 3.3.2. Generalization of Cognitive Improvements to Other Non-Trained Cognitive Functions

An important question is whether CRT generalizes improvements to other non-trained functions, which patients need in their daily lives. These effects appear to be modest, even when only studies are considered, which provided an embedded format within a psychiatric rehabilitation setting. The meta-analysis by Vita et al. (2021) showed overall low effect sizes for non-trained functions, such as global function (*d* = 0.22), social cognition (*d* = 0.24), and the lowest effect on overall psychiatric symptoms (*d* = 0.14) [33]. In general, the best results regarding transfer of skills achieved with CRT are seen when CRT is delivered within a psychiatric rehabilitation setting.

#### 3.3.3. Predictors of Cognitive Response

As with other interventions, not all patients respond to CRT. Predictor studies have identified a number of response predictors. The meta-analysis by Vita et al. (2021) identified fewer years of education and lower global functioning, lower premorbid IQ, and higher symptom severity level at baseline as predictors of better response to CRT [33]. Global functioning, unlike global cognition, is the degree to which the symptoms of schizophrenia affect social, occupational, and psychological functioning. Thus, global functioning may be an important target to measure before CRT. These predictors are to some degree unexpected, given the level of cognitive tasks participants must practice. One explanation may be that there is more room for improvement for patients who start out at a lower level of cognitive functioning. In fact, some studies have found different predictors, such as better baseline speed of processing and attention, better working memory, younger age and better education level predicting better response to CRT [37]. Clearly, a prerequisite for optimal information intake during CRT exercises is the ability to be attentive and to process information [37].

#### 3.3.4. Social Cognition and Social Functioning as Trained Outcomes

Along with the cognitive domains targeted, CRT can be used to augment and target several other outcomes. Specifically, social cognition and social functioning have gained increased attention due to their strong association with real-world outcomes [47].

Social cognition improved in several studies that embedded social cognition training within the CRT program, used an adjunctive social cognition or a social skills training program alongside CRT, or did not target social cognition at all. Three studies used REHACOP, which embeds social cognition training within the CRT program. The authors found moderate to large effect sizes in social cognition (Theory of Mind: η^2^_p_ = 0.148 [19] and η^2^_p_ = 0.293 [30], social perception: η^2^_p_ = 0.082, emotion perception: η^2^_p_ = 0.071, managing emotions: η^2^_p_ = 0.066 [19], and emotion processing: η^2^_p_ = 0.137 [30]). Social cognition was improved in two studies that implemented an adjunctive social cognition training program to CRT [10,21] with effect sizes ranging from 0.73 to 1.27. One study [8] randomized participants to either the Training of Affect Recognition (TAR) program or to CRT and found an improvement in prosodic affect recognition with a large effect size (*d* = 0.89), ToM (*d* = 1.14), social competence (*d* = 0.75), and a medium effect in the Social and Occupational Functioning Scale (*d* = 0.58). Three studies [16,24,25] that did not include any social cognitive training found significant effects on social cognition with effect sizes ranging from 0.50 to 0.91. Jahshan et al. (2019) also did not include a social cognitive training program but reported a significant improvement in social cognition (*p* = 0.008) [27]. One study [23] did not report an effect size but observed a significant improvement in social cognition in favor of CRT, plus vocational job training at a 12-month follow-up (*p* = 0.006). Not only did the CRT group in this study improve in social cognition, but they also found individuals in the CRT condition to work significantly more hours (*p* = 0.020) at their place of employment [23].

In terms of targeting social functioning, one study showed improvements in functioning for the CRT condition in social competence (*d* = 0.56) [15]. Kurtz et al. (2015) showed that the CRT group improved more in empathy compared to the control group with a medium effect size (*d* = 0.67). Measured by the Personal and Social Performance scale, one study [10] found a significant improvement in social functioning with a medium effect size (*d =* 0.47) and another [19] found a significant improvement in the UCSD Performance-Based Skills Assessment with a η^2^_p_ = 0.154 effect size. Of note, the REHACOP program has shown improvements in social functioning using a multi-dimensional approach, which went beyond CRT. This integrative program targets cognition, social cognition, and functioning, and reciprocally boosts the effect of treatment. Three studies [15,28,30] implementing REHACOP showed improvements in social functioning (social competence: *d* = 0.56, vocational outcome: *d* = 0.47, family contact: *d* = 0.50 [15], global functioning: η^2^_p_ = 0.154 [19] and η^2^_p_ = 0.253 [30], and functional competence: η^2^_p_ = 0.154 [19]). Given the vast evidence of the benefits of combining different cognitive and social cognition training approaches and the known association between social cognition and functional outcomes [19], integrative programs have the potential to ameliorate the transfer of cognitive gains to real world outcomes.

## 4. Discussion

Our review of the effectiveness of CRT for the treatment of cognitive symptoms of schizophrenia confirms results reported in three previous meta-analyses [1,6,33]. While the effect sizes of these cognitive results are of medium strength, they are robust and have been repeatedly confirmed. The strongest effects are seen in global cognition and in the cognitive domains of verbal learning and working memory, followed by lesser effects on attention and processing speed and minimal effects on problem solving and reasoning, establishing CRT as a valid treatment for cognitive dysfunction in patients with schizophrenia. The studies reviewed reflect a wide range of patient samples in terms of illness phase, age, and level of function of patients, and in terms of in- vs outpatient status. We also found that the effectiveness of CRT is significantly moderated by the four core elements of CRT, proposed by the expert working group [5]: (1) the presence of a trained therapist; (2) repeated practice of cognitive exercises; (3) structured development of cognitive strategies and (4) the use of techniques for transfer to the real world.

The effects of CRT are more robust if conducted with a trained CRT therapist, either on an individual level or in a group setting with a bridging group developing cognitive strategies and facilitating the transfer of learned cognitive skills to everyday life. Cognitive exercises should be practiced over many sessions with increasing levels of difficulty, adapted to the cognitive level of the participant. Results appear not to differ depending on the methodology, which can be paper-and-pencil-based or use a computerized program. However, there are no definite studies to inform on the precise duration of the treatment, nor on the frequency of CRT sessions. All three meta-analyses [1,6,33] find that the type of CRT does not appear to affect the cognitive outcomes of participants. Mechanistically, CRT approaches have been categorized as either top-down or bottom-up approaches. Both types of approaches appear to reach the same cognitive results but differ in terms of their respective outcome measures.

Another core element of CRT is the provision of a structured system of cognitive strategies. CRT studies, which include a focus on strategy development and elements of metacognitive training tend to show better results. Effective CRT studies do include the availability of techniques for the transfer to the real world. It appears that the most effective transfer technique is when CRT is embedded in a psychiatric rehabilitation program, which provides opportunities for participants for the transfer of cognitive skills to everyday functioning. CRT will aid in the gains from psychiatric rehabilitation, which in turn can boost the effectiveness of CRT. Another tool for the transfer of cognitive gains into real world function is the inclusion of bridging groups in CRT. Participants can practice new skills, which may aid the generalizability of CRT to other non-trained functions.

Our review found that results on the predictors of response to CRT were heterogenous. While the meta-analysis of Vita et al. (2021) found unexpectedly that less education and lower global functioning, lower premorbid IQ and higher symptom severity were predictors of better response to CRT [33], some studies have found that younger age and better baseline cognitive function predicted better outcome. Our conclusion is that CRT can be beneficial both for more ill inpatients as well as for higher-functioning outpatients.

The effects of CRT on other function domains are weaker, as seen for the effects on social functions and social cognition. These outcomes are evidently more distant to cognitive outcomes and are typically not being trained by CRT. However, one could expect to see an effect by CRT, as cognitive functions are necessary to perceive and correctly identify emotion expressions in others and are required to function in real world settings.

However, effect sizes for social cognition and social function were low. Studies that also included a training intervention on social cognition or on social function achieved a higher effect size. We conclude that an effective intervention to address these other domains would be a combination of CRT and a social cognition program.

Interestingly, the role of antipsychotic concomitant medication during CRT is rarely examined. While most participants in all CRT studies are taking antipsychotic medications, there are few studies examining the specific type of antipsychotics used or any dose effects, which may reduce the effects of CRT. There has been interest, however, in the effects of concomitant anticholinergic medications, which are often co-prescribed to treat extrapyramidal symptoms in patients with schizophrenia. Vinogradov et al. (2009) examined the deleterious anticholinergic burden on cognitive functions and found significant negative effects during CRT [35].

### 4.1. Barriers and Future Developments

It is surprising that CRT has not moved more forcefully from its use in research settings to common clinical routine use. Several factors may have limited its full introduction in the US. First, CRT is not a billable intervention by most insurance carriers, as pharmacotherapy and psychotherapy are, making it more difficult for clinicians and mental health providers to be reimbursed for this service. Its limited transfer to real world functions may be another barrier hindering its routine implementation. There is limited data on the durability of the achieved gains in cognitive functions after CRT. It is not clear whether “booster” sessions may be needed after a certain time after completion of CRT. There is a lack of formal education of CRT therapists in training programs for mental health providers, making it more difficult in finding well-trained CRT providers. Finally, there may be less awareness among clinical providers of the cognitive deficits in their patients, as they are dealing with the very visible positive symptoms of schizophrenia, while cognitive deficits are more discrete.

There are promising developments in CRT, which may help its future dissemination to a larger user base. There are several pro-cognitive drugs in development which may eventually provide significant augmentation strategies to CRT. Augmentation effects with tDCS and other neuro-modulatory interventions are being explored. Finally, studies are exploring further bottom-up approaches by training auditory and visual perception in the context of CRT [34,36].

### 4.2. Limitations

There are several limitations inherent in our review. The current paper did not strictly follow PRISMA systematic review procedures, which limits the applicability and replicability of our findings. Potential methodological and publication biases should also be considered. While we did not formally evaluate the quality of evidence provided from the included articles, our inclusion criteria were geared to select high-quality studies and eliminated a high number of CRT studies in our search. Lastly, due to the small sample of articles evaluated, there may be a selection bias that lacks comprehensiveness and representation of the extant research on CRT in schizophrenia.

## 5. Conclusions

Our review confirms results reported in previous meta-analyses of the efficacy of CRT for individuals suffering from schizophrenia [1,6,33]. Effect sizes of these cognitive results are of medium strength, with the strongest effects in global cognition and in the cognitive domains of verbal learning and working memory. The effects of CRT are more robust if conducted with a trained CRT therapist, in a group setting with a bridging group or embedded in a general rehabilitation setting, which facilitates the transfer of learned cognitive skills to everyday life. Remaining questions that need further study are the persistence of gains achieved with CRT, the facilitation of transfer of cognitive gains to real world function, and the transfer of CRT from research settings to general clinical practice. A promising future development is the application of synergistic effects of pro-cognitive medications with CRT interventions.

## Figures and Tables

**Table 1:** Summary of articles included in the results.

Study	Design	Active (A) and Control/Comparator (C)	Number of Participants	Duration (wks)	Target Outcome and Measures	Results
d’Amato et al. (2011) [7]	Single-blind RCT	A: RehaComC: Waitlist	77	7 weeks plus 12-week follow-up	Neurocognition: Cogtest Battery Test Community	Verbal Learning: *d* = 1.55 Attention: *d* = 0.42, *d* = 0.48 Verbal Memory: *d* = 0.52 Working Memory: *d* = 0.41
					Functioning: Social Autonomy Scale	Functioning: ns
Wolwer et al. (2011) [8]	Single-blind RCT	A: Training of Affect Recognition C: CogPack	38	6	Social Cognition: Pictures of Facial Affect; Geneva Vocal Emotion Expression Stimulus; Theory of Mind Questionnaire; Role-play task	Prosodic affect recognition: *d* = 0.89 Theory of Mind: *d* = 1.14
					Social Functioning: Social and Occupational Functioning Assessment Scale	Social competence: *d* = 0.75 Social functioning: *d* = 0.58
Gharaeipour & Scott (2012) [9]	Single-blind RCT	A: Adapted CRT C: Group supportive therapy	42	8	Neurocognition: Rey Auditory Verbal Learning Test; Wisconsin Card Sorting Test; Auditory Consonant Trigrams; Rey-Osterrieth Complex Figure Test; Trail Making Test A and B	Cognitive composite: *d* = 0.37 Attention: *p* = 0.044 Processing Speed: *p* = 0.013 Visual Learning and Memory: *p* = 0.020, *p* = 0.014 Executive Function: 0.039 Verbal Working Memory: *p* = 0.023 Verbal Learning: *p* = 0.020
Lindenmayer et al. (2012) [10]	RCT	A: CogPack plus Mind Reading: Interactive Guide to Emotions (MRIGE) C: CogPack	59	12	Neurocognition: MATRICS Consensus Cognitive Battery	Cognitive Composite: *d* = 0.30 Processing Speed: *d* = 0.32 Attention/Vigilance: *d* = 0.26 Working Memory: *d* = 0.32
					Social Cognition: Facial Emotion Identification Test; Facial Emotion Discrimination Test; Mayor-Salovey-Caruso Emotional Intelligence Test	Social Cognition: *d* = 0.73 Emotion Perception: *d* = 1.24, *d* = 1.27
					Social Functioning: Personal and Social Performance Scale	Social Functioning: *d* = 0.47
Rass et al. (2012) [11]	Three-arm parallel, single-blind RCT	A: Posit Science C: Watch movies/television C: TAU	44	10 weeks plus 20-week follow-up	Neurocognition: MATRICS Consensus Cognitive Battery	Global Cognition: ns
Keefe et al. (2012) [12]	Single-blind randomized-controlled feasibility and pilot trial	A: Posit Science Brain Fitness C: Computer games and healthy lifestyles group	53	12	Neurocognition: MATRICS Consensus Cognitive Battery	Global Cognition: ns
Lu et al. (2012) [13]	RCT	A: Frontal/Executive Program C: TAU	126	12	Neurocognition: Wisconsin Card Sorting Test	Cognitive Functioning: *p* = 0.019
					Social Functioning: Scale of Social Skills of chronic schizophrenia Inpatients	Functioning: ns
Hubacher et al. (2013) [14]	Randomized-controlled pilot trial	A: Brainstim C: Waitlist	29	4	Neurocognition: Verbal Fluency Test; Selective Reminding Test; Spatial Recall Test; Symbol Digit Modalities Test; Test Battery for Attention Performance; Wechsler Memory Scale-Revisied; Paced Auditory Serial Addition Test	Verbal Working Memory: *d* = 1.04
Sánchez et al. (2014) [15]	Single-blind RCT	A: REHACOP C: Drawing, reading, and constructing objects	84	12	Neurocognition: Wechsler General Intelligence Scale-III; Hopkins Verbal Learning Test; Semantic and Phonological Fluency Subtests from Barcelona Test	Processing speed: *d* = 0.63 Working Memory: *d* = 0.88 Verbal Learning: *d* = 0.88
					Global Functioning: Global Assessment of Functioning; Clinical Global Impressions; Disability Assessment Schedule-World Health Organization	Social Competence: *d* = 0.56 Vocational Outcome: *d* = 0.47 Family Contact: *d* = 0.50
Mendella et al. (2015) [16]	Randomized-controlled pilot trial	A: Compensatory Cognitive Training C: TAU	27	12	Neurocognition: MATRICS Consensus Cognitive Battery	Cognitive Composite: η^2^_p_ = 0.350 Processing Speed: η^2^_p_ = 0.178
					Social Cognition: Mayor-Salovey-Caruso Emotional Intelligence Test	Social Cognition: η^2^_p_ = 0.170
					Global Functioning: University of California, San Diego Performance Based Skill Assessment-Brief Version	Functioning: ns
Kurtz et al. (2015) [17]	Single-blind RCT	A: CogRem plus Social Skills Training C: Computer games plus Social Skills Training	56	23	Neurocognition: Wechsler Adult Intelligence Scale-III/IV; Penn Continuous Performance Test; California Verbal Learning Test	Attention and Working Memory: *d* = 0.46
					Social Functioning: Social Skills Performance Assessment; Quality of Life Scale-Brief	Empathy: *d* = 0.67
Matsuda et al. (2016) [18]	Single-blind randomized-controlled feasibility trial	A: Japanese Cognitive Rehabilitation Programme for Schizophrenia C: TAU	62	12	Neurocognition: Brief Assessment of Cognition in Schizophrenia-Japanese	Cognitive Composite: *p* = 0.047 Verbal Memory: *p* = 0.008
					Social Functioning: Life Assessment Scale for Mentally Ill	Functioning: ns
Peña et al. (2016) [19]	Single-blind, parallel-group RCT	A: REHACOP C: Occupational group activities	111	16	Neurocognition: Hopkins Verbal Learning Test; Stroop Test; Wechsler Adult Intelligence Scale-III; Accentuation Reading Test Based Skill Assessment	Cognitive Composite: η^2^_p_ = 0.138
					Social Cognition: Happé Test; Mayer-Salovery-Caruso Emotional Intelligence Test-Spanish; Situational Feature Recognition Test; Attributional Style Questionnaire	Theory of Mind: η^2^_p_ = 0.148 Social Perception: η^2^_p_ = 0.082 Emotion Perception: η^2^_p_ = 0.071 Managing Emotions: η^2^_p_ = 0.066
					Global Functioning: Global Assessment of Functioning; University of California, San Diego Performance	Functional Competence: η^2^_p_ = 0.154 Global Functioning: η^2^_p_ = 0.154
Fan et al. (2017) [20]	Single-blind RCT	A: Computerized CRT C: TAU	23	8	Neurocognition: MATRICS Consensus Cognitive Battery	Processing Speed: *p* = 0.01
Lindenmayer et al. (2018) [21]	Single-blind, parallel-group RCT	A: CogPack or Brain Fitness plus MRIGE C: CogPack or Brain Fitness alone	78	12	Neurocognition: MATRICS Consensus Cognitive Battery	Cognitive Composite: *d* = 2.37 Visual Learning: *d* = 1.51 Working Memory: *d* = 1.50 Processing Speed: *d* = 1.93
					Social Cognition: Facial Emotion Identification Test; Facial Emotion Discrimination Test; Dynamic Social Cognition Battery; Penn Emotion Recognition Task	Emotion Recognition: *d* = 0.85 Emotion Perception: *d* = 1.21
					Global Functioning: University of California, San Diego Performance Based Skill Assessment-Brief Version	Functioning: ns
Bryce et al. (2018) [22]	Parallel, single-blind RCT	A: CogPack C: Computer games	56	10 weeks plus 12-week follow-up	Neurocognition: MATRICS Consensus Cognitive Battery	Cognitive Composite: *d* = 0.68
					Global Functioning: Independent Living Skills Survey-Self Report	Functioning: ns
Kukla et al. (2018) [23]	Single-blind RCT	A: Posit Science Brain Fitness and Insight plus Cognitive Behavioral Therapy (CBT) C: CBT	75	26 weeks plus 48-week follow-up	Neurocognition: MATRICS Consensus Cognitive Battery	Cognitive Composite: *p* = 0.002 Verbal Learning: *p* = 0.003
					Social Cognition: Mayer-Salovery-Caruso Emotional Intelligence Test	Social Cognition: *p* = 0.006
					Work Outcome: Work Behavior Inventory	Worked more hours: *p* = 0.02
Cassetta et al. (2018) [24]	Three-arm parallel, double-blind RCT	A: BrainGymmer: Working Memory A: BrainGymmer: Processing Speed C: TAU	71	10	Neurocognition: N-Back; Maintenance and Manipulation Task; Digit Span; Delis-Kaplan Executive Function System	Working Memory: ns Processing Speed: η^2^_p_ = 0.107 Executive Function: η^2^_p_ = 0.112 and 0.132
					Social Cognition: Hinting Task; Geneva Emotion Recognition Test	Social Cognition: η^2^_p_ = 0.146
					Global Functioning: Cognitive Failures Questionnaire; University of California, San Diego Performance Based Skill Assessment-Brief Version; Social and Occupational Functioning Assessment Scale	Functioning: η^2^_p_ = 0.139
Contreras et al. (2018) [25]	Single-blind RCT	A: CogPack plus Visual Processing Training C: CogPack alone	20	10	Neurocognition: MATRICS Consensus Cognitive Battery	Visual Learning: *d* = 0.88 Working Memory: *d* = 0.44
					Social Cognition: Mayor-Salovey-Caruso Emotional Intelligence Test	Social Cognition: *d* = 0.50
Ventura et al. (2019) [26]	RCT	A: Adapted Neuropsychological Educational Approach to Remediation (NEAR) and Neurocognitive Enhancement Therapy (NET) C: Healthy Behaviors Training	80	24 weeks plus 24 weeks of booster sessions	Social Functioning: UCLA Social Attainment Survey	Social Functioning: *p* = 0.05
Jahshan et al. (2019) [27]	Three-arm parallel, RCT	A: Brain Fitness A: CogPack C: Sporcle Computer games	99	12	Neurocognition: MATRICS Consensus Cognitive Battery	Cognitive Composite: ns
					Social Cognition: Mayor-Salovey-Caruso Emotional Intelligence Test	Social Cognition: *p* = 0.008
					Global Functioning: University of California, San Diego Performance Based Skill Assessment	Functioning: ns
Linke et al. (2019) [28]	RCT	A: CogPack C: Relaxation training, integration games, and lectures	66	6	Neurocognition: MATRICS Consensus Cognitive Battery	Cognitive Composite: ns
					Social Cognition: Mayor-Salovey-Caruso Emotional Intelligence Test	Social Cognition: ns
					Global Functioning: Global Assessment of Functioning	Functioning: ns
Molina et al. (2020) [29]	Parallel-group RCT	A: BrainHQ C: Computer games	42	10–12	Neurocognition: MATRICS Consensus Cognitive Battery	Cognitive Composite: *R^2^* = 0.31 Attention/Vigilance: *R^2^* = 0.18 Working Memory: *R^2^* = 0.17
Sampedro et al. (2021) [30]	Parallel, single-blind RCT	A: REHACOP C: Occupational group activities	94	20	Neurocognition: Modified Wisconsin Card Sorting Test; Wechsler Adult Intelligence Scale-III; Stroop Color and Word Test; Hopkins Verbal Learning Test	Processing Speed: η^2^_p_ = 0.190 Working Memory: η^2^_p_ = 0.074 Verbal Memory: η^2^_p_ = 0.166
					Social Cognition: Happé Test; Social Attribution Task-Multiple Choice; Bell Lysaker Emotion Recognition Test	Theory of Mind: η^2^_p_ = 0.293 Emotion Processing: η^2^_p_ = 0.137
					Global Functioning: University of California, San Diego Performance Based Skill Assessment; Social Functioning Scale	Functioning: η^2^_p_ = 0.253
Hatami et al. (2021) [31]	Single-blind RCT	A: CogPack C: TAU	62	4–5	Neurocognition: Cambridge Neuropsychological Test Automated Battery	Visual Memory: *d* = 0.14–0.71
					Global Functioning: Global Assessment of Functioning	Functioning: *p* = 0.034
Zhu et al. (2021) [32]	Three-arm parallel, single-blind RCT	A: CogSMARTA: CogSMART plus Medication Self-Management Skills Training C: TAU	72	4	Neurocognition: Brief Assessment of Cognition in Schizophrenia	Cognitive Composite: η^2^_p_ = 0.275 Verbal Learning: η^2^_p_ = 0.373
					Medication Adherence: Medication Adherence Questionnaire	Medication Adherence: η^2^_p_ = 0.127

Legend of Abbreviations: wks, Weeks; CRT, Cognitive Remediation Training; RCT, Randomized-Controlled Trial; TAU, Treatment as Usual; *d*, Cohen’s d Effect Size; ns, Non-Significant; *p*, Statistical Significance p-value; η^2^_p_, Partial eta-Squared; *R*^2^, r-squared Coefficient of Determination. Note. Only statistically significant findings are presented in the results column. When effect sizes were not given, p-values were included. Secondary outcome targets and results that were out of the scope of this review were omitted.

## Data Availability

No new data were created or analyzed in this study. Data sharing is not applicable to this article.
